# Duodenoscope-Associated Infections beyond the Elevator Channel: Alternative Causes for Difficult Reprocessing

**DOI:** 10.3390/molecules24122343

**Published:** 2019-06-25

**Authors:** Gheorghe G. Balan, Irina Rosca, Elena-Laura Ursu, Adrian Fifere, Cristian-Dragos Varganici, Florica Doroftei, Ioana-Andreea Turin-Moleavin, Vasile Sandru, Gabriel Constantinescu, Daniel Timofte, Gabriela Stefanescu, Anca Trifan, Catalin Victor Sfarti

**Affiliations:** 1 Department of Gastroenterology, Faculty of Medicine, “Grigore T. Popa” University of Medicine and Pharmacy, 16 University Street, 700115 Iași, Romania; balan.gheo@me.com (G.G.B.); dantimofte@yahoo.com (D.T.); gabriela.stefanescu@gmail.com (G.S.); ancatrifan@yahoo.com (A.T.); cvsfarti@gmail.com (C.V.S.); 2 Centre of Advanced Research in Bionanoconjugates and Biopolymers (IntelCentru), ‘‘Petru Poni’’ Institute of Macromolecular Chemistry, 41A Grigore Ghica Vodă Alley, 700487 Iași, Romania; ursu.laura@icmpp.ro (E.-L.U.); fifere@icmpp.ro (A.F.); varganici.cristian@icmpp.ro (C.-D.V.); florica.doroftei@icmpp.ro (F.D.); moleavin.ioana@icmpp.ro (I.-A.T.-M.); 3 Department of Gastroenterology Research, Clinical Emergency Hospital of Bucharest, 8 Calea Floreasca, 014461 Bucharest, Romania; drsandruvasile@gmail.com (V.S.); gabrielconstantinescu63@gmail.com (G.C.); 4 Internal Medicine Department, Faculty of Medicine, “Carol Davila” University of Medicine and Pharmacy, 37 Dionisie Lupu Street, 030167 Bucharest, Romania

**Keywords:** nosocomial infections, endoscopic retrograde cholangiopancreatography, reprocessing, carbapenem-resistant enterobacteriaceae

## Abstract

Objectives: Duodenoscopes have been widely used for both diagnostic and therapeutic endoscopic retrograde cholangiopancreatography (ERCP) procedures, but recently, numerous outbreaks of multidrug-resistant organisms (MDRO) infections have been reported which has led to extensive research for their possible causes. Consequently, the aim of this study is to search for possible duodenoscope surface damages that could provide an alternative and plausible source of infections. Materials and Methods: In order to assess both outer and inner surfaces, a duodenoscope was dismantled and samples were taken from the outer resin polymer and from the air/water, elevator, and working (biopsy) channels that were characterized by FTIR, DSC, TGA, AFM, SEM techniques and the antimicrobial activity were tested. Results: Alterations were noticed on both the coating and working channel polymers, with external alterations increasing progressively from the proximal sample to the distal sample near the tip of the scope. However, the results showed that the coating surface was still efficient against bacterial adhesion. Changes in surface texture and also morphological changes were shown. Conclusions: The study describes the impact of routine procedural use and reprocessing cycles on the duodenoscope, showing that these may possibly make it susceptible to bacterial contamination and MDRO biofilm formation due to difficult reprocessing of the altered surfaces.

## 1. Introduction

Despite the fact that the first duodenoscope-transmitted infection was described more than 30 years ago by Allen et al. [1], multiple recent reports associate multidrug-resistant organisms (MDRO) with duodenoscope-transmitted infections during endoscopic retrograde cholangiopancreatography (ERCP), which has led to a reassessment of current reprocessing standards and infection control practices [2] and subsequently to extensive research for the possible causes of such infections. Particularly, MDROs, such as carbapenem-resistant enterobacteriaceae (CRE), were associated with ERCP-related nosocomial clusters due mainly to difficulties in the adequate cleaning of the elevator channel and recess [3], which are independent of potential breaches in the reprocessing standards [4,5]. Therefore, the United States Food and Drug Administration (FDA) repeatedly advises health care professionals to strictly follow the manufacturer issued duodenoscope reprocessing protocols emphasizing the need for double reprocessing cycles backed by surveillance protocols like the “culture and hold” policy that would permit constant high level disinfection (HLD) monitoring prior to use [2,6]. Potential alternatives to HLD are sterilization with ethylene oxide gas or liquid chemical sterilant [2].

The development of a multidrug-resistant biofilm is thought to be triggered by repeated bacterial contamination of the duodenoscope parts that are difficult to clean or even sealed [5]. During the last years, numerous outbreaks of duodenoscope-associated transmission of multidrug-resistant bacteria have been reported worldwide, with *Staphylococcus aureus*, *Klebsiella pneumonia*, *Enterococcus faecalis*, *Escherichia coli*, and *Pseudomonas aeruginosa* being the most frequently reported bacteria to cause contaminations and duodenoscope-transmitted infections, especially because of their ability to form biofilms [7]. Hence, manufacturers, such as Olympus America, have repeatedly announced sustained arrangements for the return of scope devices for elevator replacement that would allow consistency with the FDA cleared protocols [8].

Moreover, manufacturers have developed new duodenoscope models that either ensure proper sealing of the less accessible elevator channel (OLYMPUS GmbH), or create detachable and autoclavable (KARL STORZ, Germany) or even single-use disposable elevator devices (PENTAX Medical, DEC™).

Although such new measures need further real-life confirmation studies, MDRO duodenoscope-related infections are still a constant healthcare issue [9,10,11] broadly characterizing all duodenoscope models involved, and therefore raising questions about other possible causes for MDRO harboring and transmission. Furthermore, recent data sustain the idea that most of the inert surfaces of the scopes become more susceptible to contamination because of significant deterioration, due to repeated daily use, which leads to the harboring of resistant bioburden [12,13]. MDROs have the capacity to form biofilms that are resistant to both physical cleaning and chemical disinfection [14] and such biofilm formation is promoted by the difficulty of accessing surface reprocessing, and also by surface defects from manufacturing or secondary to physical damage due to passing forceps or routine instrument handling [15,16].

Consequently, the aim of our study was to describe possible scope surface damages secondary to routine wear that could provide an alternative cause for duodenoscopes being less amenable to proper reprocessing and clearance of bioburden.

## 2. Results and Discussion

### 2.1. Fourier Transform Infrared Spectroscopy (FT-IR)

**Figure 1:**
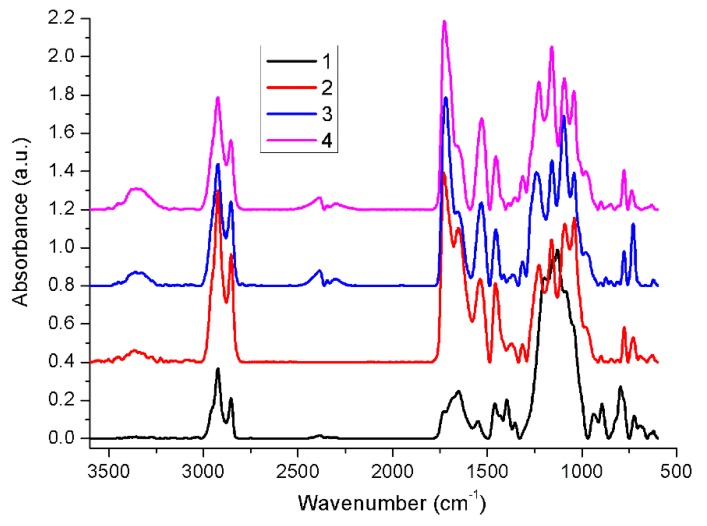
FTIR spectra of the studied samples 1–4.

Figure 1 presents the FT-IR spectra of the samples with a higher degree of wear in the reverse order of their numbering. From the fourth to first sample, significant differences occur in the region 1720–1730 cm^−1^, assigned to carbonyl vibrations, by a major decrease in the carbonyl band intensity. This occurs in such a way that the intensity ratio between the carbonyl group band and the amide specific band 1652 cm^−1^ are decreasing from the fourth to the first sample.

Some authors associated this event with a change in crosslinking density [17], which confirms the results obtained by thermal analysis. In addition, a lowering ratio between the intensity of the bands from the 1600–1760 cm^−1^ region going from the fourth to the first sample, associated with C=O and C=N vibrations, and 1040–1200 cm^−1^ region, generally associated with C–O, C–O–C, C–N vibrations [18,19] suggest chemical bonds rearrangements. Since the FT-IR spectra reveal substantial changes in the vibrational band intensities ratio of different chemical bonds from the fourth to the first sample, FT-IR results could explain the morphological changes that determined the variation in thermal properties observed in the DSC and TGA assays.

### 2.2. Thermal Behavior

**Figure 2:**
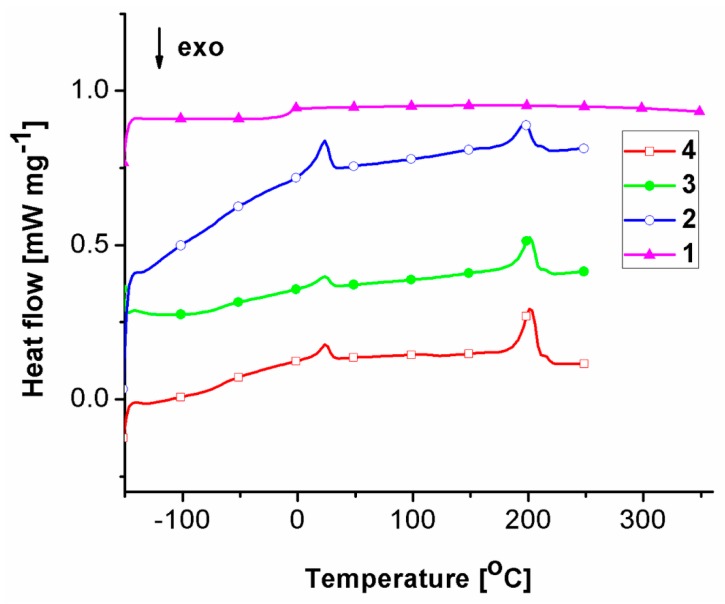
DSC second scans of the studied samples.

The thermal behavior of the studied samples was assessed with the aid of DSC and TGA measurements, since the heat flow generated transitions and thermal decomposition profiles are very useful in establishing general differences in materials aging pathways and/or patterns. Figure 2 shows the DSC second heating scans and their characteristics, recorded up to the onset temperature of thermal degradation.

**Figure 3:**
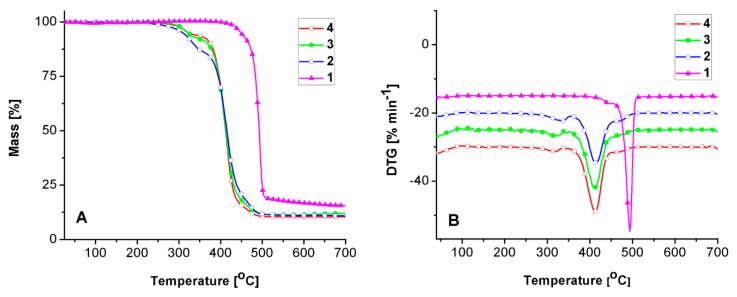
(**A**) TGA and (**B**) DTG curves of the studied samples.

Figure 2 and Table 1 show that the initial fourth sample exhibits a T_g_ domain at −67 °C and two melting profiles, one registered at a lower temperature (T_m_ = 24 °C) and one at a higher temperature (T_m_ = 202 °C). By analyzing the DSC data, one observes a general decreasing trend in melting enthalpy values, and thus in crystallinity degree. Samples three, two and one witness an increase in T_g_ values to −65 °C, −63 °C, and −5 °C. This aspect, correlated with the significant increase in thermal stability (Figure 3, Table 2), from 328 °C (sample four), 323 °C (sample three) and 309 °C (sample two) to 445 °C (sample one), shows that the aging process primarily induces crosslinking. Hence the aging process of the material occurs through a decrease in crystallinity and chemical crosslinking. There were no noticeable differences between the residue masses of samples four, three, and two left at the end of the thermal degradation processes (10.33–11.43%) (Table 2). Sample one exhibited the highest crosslinking, due to the disappearance of the melting profiles and the very high T_g_ increase (T_g_ = −5 °C) as compared with that of sample four (T_g_ = −67 °C). This aspect is also reflected in the differences in thermal degradation profiles, sample one manifesting the highest thermal stability (445 °C) and residue content (15.19%). Furthermore, the first derivative (DTG) curve peaks indicate three stages of thermal degradation for all the samples, except sample one. For sample one the first decomposition stage, most probably attributed to initiation of thermal degradation through scission of weak linkages, disappears leaving only the second stage, of only 8.25% mass loss, and the third stage, both at significantly higher onset temperatures. This is an indication that the first sample tends to behave as a single system [20].

### 2.3. Antimicrobial Activity

In all the cases, the samples displayed antibacterial properties and did not allow bacterial strains adhesion and growth, There were no differences between the Gram-positive (*S. aureus*) and the Gram-negative (*E. coli*) strains and after 24 h of incubation just a few colonies were found in the PCA plates (as compared with the control) suggesting that the antibacterial properties of the endoscope polymer are preserved, as proven also by their antibacterial effectiveness with R > 2 calculated according to JIS Z2801:2000 specifications [21]. As shown in Table 3, only the distal sample (first sample) was slightly less effective against both bacterial strains, however, their antibacterial effectiveness was still high. An important acknowledgement in this respect is that the duration of the experimental inoculation and incubation exceeds the usual per-procedure exposure to gut bacteria. Hence, an ERCP procedure usually lasts between 20 min and 1.5 h, afterwards duodenoscopes are pre-cleaned at bedside using enzymatic solutions and subsequently reprocessed following the manufacturer issued protocols. Nevertheless, the same duodenoscope tends to be used for up to five procedures daily in high volume centers, each procedure involving continuous aspiration of gut fluid and tissue-to-duodenoscope friction.

### 2.4. Atomic Force Microscopy (AFM)

#### 2.4.1. Morphological Characterization

**Figure 4:**
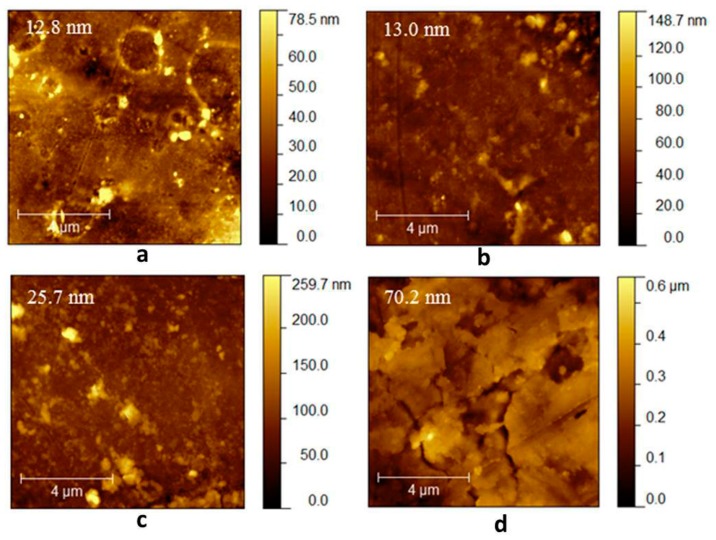
AFM topographic images for the duodenoscope samples: **a**—1st sample, **b**—2nd sample, **c**—3rd sample, **d**—4th sample.

Figure 4 presents AFM topographic images of the four duodenoscope samples. The duodenoscope surface was imaged at five randomly chosen positions from which the root-mean-square roughness (R_q_) was calculated. The samples are characterized by an inhomogeneous morphology and even microcracks are observed. The roughness average value varied from 12.8 nm for the fourth sample to 70.2 nm for the first sample, suggesting that the intense usage of the proximal part of the duodenoscope caused, in time, the polymer usage. The results are consistent with our preliminary optical analysis study carried out on similar duodenoscopes [22].

#### 2.4.2. Evaluation of Biofilm Formation on Duodenoscope Samples

**Figure 5:**
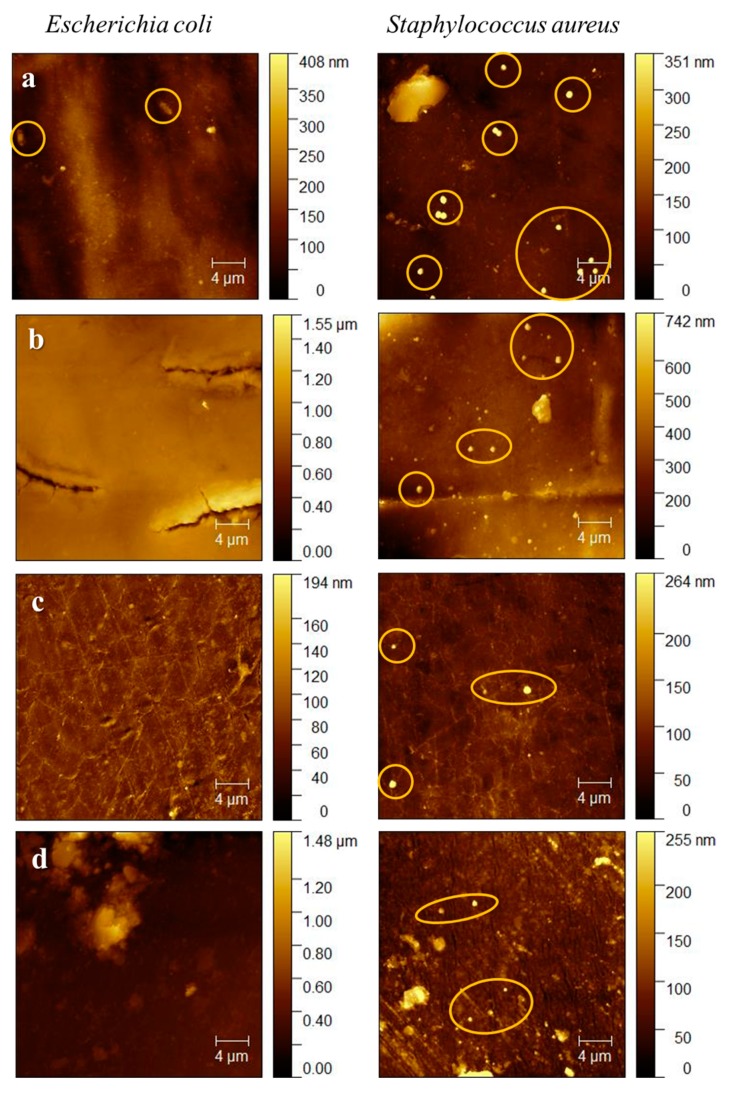
AFM topographic images for the duodenoscope samples after incubation with *Escherichia coli* and *Staphylococcus aureus*: **a**—1st sample, **b**—2nd sample, **c**—3rd sample, **d**—4th sample.

AFM was also used to determine if biofilm is formed on the duodenoscope samples after incubation with *Escherichia coli* and *Staphylococcus aureus* (Figure 5). In the case of *E. coli,* no biofilm formation is observed, however, for the distal segment, the attachment of a few rod-shaped cells (with a length of 2 µm and a width of 0.7 µm) is evident. For *S. aureus* grown on each tested sample, attached small round-shaped cells (with diameter of 0.7 µm) were individually distributed on the solid surfaces. No aggregates or colonies on tested surfaces were noticed for both bacterial strain, even though usually there were differences found between the antibacterial properties of different surfaces which were caused by the different composition of the Gram-positive and Gram-negative cell walls [23,24].

### 2.5. Scanning Electron Microscopy (SEM)

**Figure 6:**
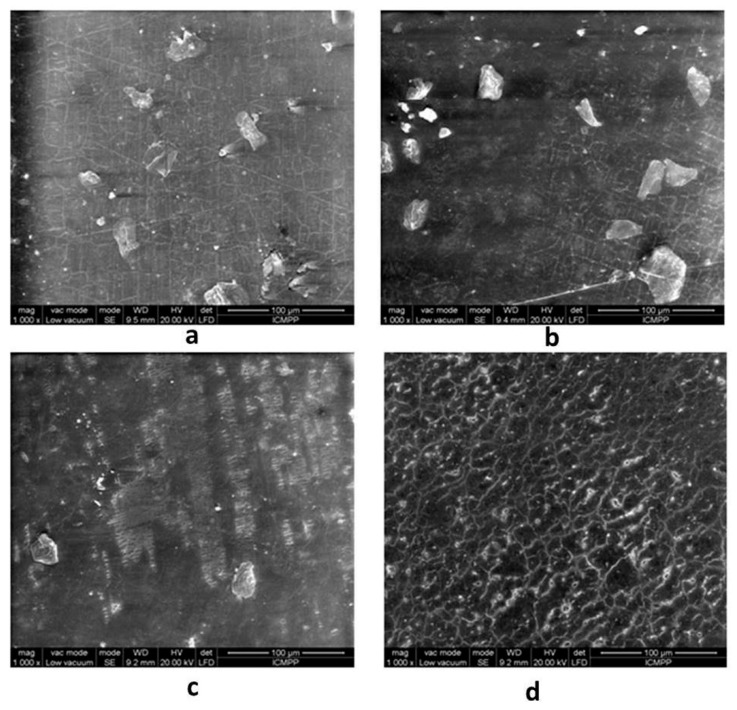
SEM micrographs of duodenoscope samples. **a**—1st sample, **b**—2nd sample, **c**—3rd sample, **d**—4th sample.

**Figure 7:**
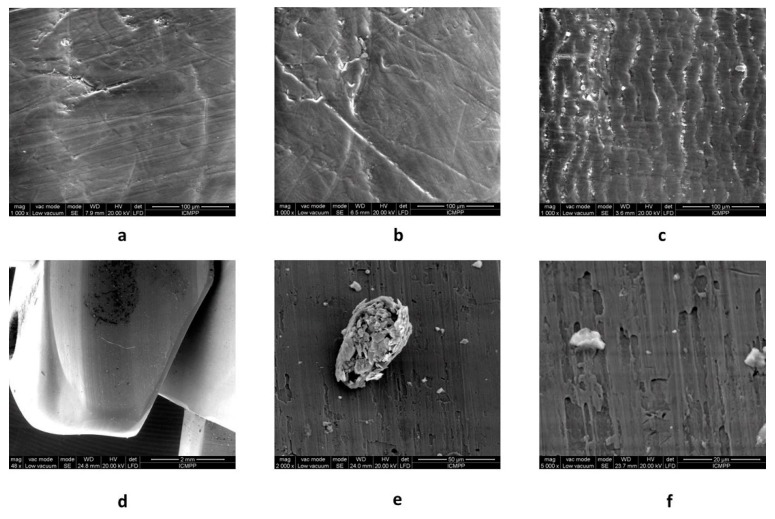
SEM micrographs of elevator and channel samples: **a**—air/water channel; **b**—elevator channel; **c**—working channel; **d**,**e**,**f**—elevator recess side.

From the SEM images (as shown in Figure 6) it is clearly observed that the coating material displays erosion and deterioration marks induced by the extensive usage and repeated reprocessing. Microscopically detached chips are also visible. Selected areas present different morphologies, resulting in different levels of deterioration. Figure 7 states for similar results inside the air/water, elevator, and working channels, and also on the elevator metallic part.

As expected, the patterns of surface alteration presented in Figure 7 are different with respect to the outer and inner (channel) surfaces. While the outer polymers, for both AFM and SEM analysis, show amorphous and irregular patterns of deterioration that could be linked to both repeated reprocessing and use, the working channel shows parallel microrecess formation mainly due to the repeated passage of instruments through the channel. A similar parallel abrasion pattern is observed on the air/water channel. Interestingly, the elevator channel shows some irregular patterns of microabrasion, in contrast to the sealed character of the channel. Nevertheless, microanalysis of the elevator recess shows both microfissures of the metallic material and remnant debris despite thorough reprocessing.

Concerning the evaluation of the macroscopic aspect of working channel polymers, a recent study allowed optical endoluminal analysis using a specially designed borescope (SteriCam, Sanovas Inc, San Rafael, CA, USA) and proved the presence of numerous scratches with adherent peel and burns, channel bulking, strains, and perforations, as well as associated solid debris and fluid residue on similarly used duodenooscopes. The authors emphasizing the need for further microscopic analysis of such findings [25]. Previous preliminary studies associated a higher degree of macroscopically evaluated working channel damages to endoscopes used in interventional procedures [26,27].

Nevertheless, as most of the up-to-date studies and FDA protocols suggest [6,28,29], breaches in duodenoscope reprocessing occur due to the difficult-to-clean design of the distal tip where, first, the opening and O-ring of the elevator channel and, secondly, the poor access to the elevator recess play a substantial role in impeding reprocessing.

On the other hand, the results of our study show that routine use of duodenoscopes causes microscopic alterations both to the outer surface of the duodenoscope coating polymers and to the inner coating of the air/water, elevator, and working (biopsy) channel. Such surface alterations were previously linked to biofilm formation [15] that protects microorganisms from the effects of thermal and chemical reprocessing agents. Therefore, it has long been shown that biofilm is visualized by electron microscopy on the inner lumen of the working and air/water channels of used gastrointestinal endoscopes [30].

The link between duodenoscope surface alterations and positive post-cleaning cultures is also sustained by the fact that, as a recent observational study shows, despite optimized and constant reprocessing protocols there are some duodenoscopes associated with a higher rate of positive cultures while some others are not [31]. Moreover, the inner channels are the ones harboring resistant bacteria (Gram-positive, spore forming) that persist even after extensive cleaning, alcoholic flush, and continuous channel purge storage conditions [11].

### 2.6. Impact on Public Health

ERCP-related infections have clearly become a worldwide issue. Moreover, infections after other various gastrointestinal endoscopic procedures were found to be substantially more common than previously thought [32], thus requiring prompt and effective administrative interventions not only on the reprocessing protocols of devices, but on the design and technology of endoscopes. In the United States, the FDA and the Centers for Disease Control and Prevention (CDC) have taken a strong position against the duodenoscope-related nosocomial infections by releasing, on February 26, 2018, new standardized protocols for duodenoscope surveillance sampling and culturing [30] that have had great visibility and impact worldwide, however the incidence and prevalence of such infections seems to remain almost unchanged [2]. Hence, healthcare facilities should clearly adopt up-to-date endoscope surveillance sampling and culturing protocols as a required and written quality policy. On the other hand, as a result of our research we outline that the industry of duodenoscope manufacturers should also actively participate by developing new materials for endoscope technology. In addition, to date, the FDA’s position is to order manufacturing companies to conduct post-market surveillance studies in order to describe the challenges of duodenoscope reprocessing in real-world settings [33]. A unified infection control policy and infection reduction strategy resulting from collaboration between health care facilities, professional societies, and institutional partners is, therefore, mandatory for mitigating infections associated with duodenoscopes. Nevertheless, as shown by our study, routine use and reprocessing cycles could microscopically impede the integrity of the polymeric materials used in duodenoscope technology, and therefore be a possible alternative cause for duodenoscope-associated infections. Such feedback should provide manufacturers with proof that there is a need for technological alternatives and innovation.

## 3. Materials and Methods

### 3.1. Duodenoscope Samples

In order to assess both outer and inner (air/water, elevator, and working channels) duodenoscope surfaces, we selected a duodenoscope from a high-volume tertiary hospital that was previously used in up to 500 ERCP procedures between 2012 and 2014. The duodenoscope was reprocessed, cultured, and quarantined, and then re-reprocessed and recultured with each reprocessing cycle performed following the manufacturer’s revised protocol. Culturing was performed by a mixed technique of channel flush, channel brushing, and elevator swabbing followed by inoculation in a liquid medium of tryptic soy broth at 36 °C for 48 h. Both culture cycles were negative. Afterwards, it was dismantled and samples from the resin polymer outer coatings were processed for analysis (first sample in contact with the distal tip, second sample at 20 cm gradation, third sample at 60 cm gradation, and fourth sample at 120 cm gradation). Samples from the air/water, elevator, and working channels were taken at 5 cm from the distal end which was the site considered most exposed to friction secondary to distal tip angulation. The elevator was detached and analyzed separately. The dismantling process was performed in a microbiologically controlled environment (Class 2 fume hood).

### 3.2. Fourier Transform Infrared Spectroscopy (FT-IR)

FT-IR spectra were recorded with a Bruker Vertex 70 FT-IR spectrometer (Billerica, Massachusetts, USA), at room temperature with a resolution of 2 cm^−1^ in the range of 500–4000 cm^−1^.

### 3.3. Differential Scanning Calorimetry (DSC)

DSC measurements were conducted on a DSC 200 F3 Maia device (Netzsch, Germany). A mass of 10 mg of each sample was heated in pierced and sealed aluminum crucibles in nitrogen atmosphere at a flow rate of 50 mL/min and a heating rate of 10 °C/min. The temperature against heat flow was recorded. The baseline was obtained by scanning the temperature domain of the experiments with an empty pan. The instrument was calibrated with indium according to standard procedures.

### 3.4. Thermogravimetric Analysis (TGA)

TGA experiments were conducted on a STA 449 F1 Jupiter device (Netzsch, Germany). The samples were heated in alumina crucibles in nitrogen atmosphere at a flow rate of 50 mL/min. A heating rate of 10 °C/min was applied.

### 3.5. Antibacterial Activity

The antimicrobial efficacy of the duodenoscope samples was investigated via a slightly modified Japanese industrial standard JIS Z2801:2000 [21] which is based on a logarithmic number of live bacteria after 24 h of incubation using a covering film to keep the thickness of the bacterial suspension. The antibacterial activity was determined against two different reference strains: *Escherichia coli* ATCC25922 and *Staphylococcus aureus* ATCC25923. The bacterial strains were refreshed in nutrient broth for 24 h at 37 °C. The test surfaces were prepared as follows: Each sample was placed in a sterile Petri dish and the bacterial inoculum was adjusted to standard 0.5 McFerland and the necessary dilutions were made to obtain 2.4 × 10^5^ CFU/mL. Then, 0.4 mL of the inoculum was instilled on the sample surface and left to incubate for 24 h at 37 °C. After incubation, the samples were rinsed repeatedly and pipetted to 1.5 mL Eppendorf tubes. The resulted suspension was serially diluted and the number of CFU’s was determined by plate counting agar (PCA) after 24 h of incubation at 37 °C. The antibacterial rate was determined using JIS Z2801:2000 specifications [21]. The standard uses a factor called R that compares, in a logarithmic scale, the number of colonies grown on the surface of the samples after 24 h of incubation as compared with the reference samples. Samples that presented a bacterial mortality higher than 99% (R > 2) were considered valid. The tested duodenoscope samples were further used in order to be scanned for biofilm formation.

### 3.6. Atomic Force Microscopy (AFM)

Images from the study samples collected from the surface of the duodenoscope were recorded in air, in tapping mode using a NTEGRA Spectra (NT-MDT, Zelenograd, Russia) instrument with a 3.1–37.6 N/m force constant cantilever of silicon nitride cantilevers (NSC10, NT-MDT, Russia). To record the biofilms, the measurements were performed in a similar fashion.

### 3.7. Scanning Electron Microscopy (SEM)

The surface morphology of the study samples was observed using a Quanta200 scanning electron microscope, (FEI Company, Oregon, USA), working in a low vacuum mode, at 20 kV with a LFD detector.

## 4. Conclusions

The present study brings new evidence regarding the assessment of duodenoscope reliability concerning the coating materials in both direct and indirect contact with living tissues, via devices manipulated through the working channel. We noticed alterations of both the coating and working channel polymers due to usage, even for a relatively small number of cases. External alterations increase progressively from the distal to the proximal sample to the elevator sample. However, the coating surface was proven to still be efficient against bacterial adhesion. Changes in terms of surface texture (roughness and cracks due to erosion, chemical resistance, and aging of material) and it was also shown that morphological changes correlated well with the variation in physical properties. Moreover, despite reprocessing and long-term quarantine the elevator harbors remnant possibly organic material suggestive for biofilm formation. All this physical evidence shows that the impact of routine procedural use and reprocessing on the scope possibly makes it susceptible to bacterial contamination and MDRO biofilm formation due to difficult reprocessing of altered surfaces.

## Figures and Tables

**Table 1:** Data evaluated from DSC measurements.

Sample	T_g_ (°C)	T_m_ (°C)		ΔH_m_ (J g^−1^)	
		Lower Profile	Upper Profile	Lower Profile	Upper Profile
4	−67	24	202	2.968	14.88
3	−65	24	201	2.218	10.55
2	−63	24	197	7.5	9.169
1	−5	–	–	–	–

T_g_—glass transition temperature; T_m_—melting temperature; ΔH_m_—melting enthalpy.

**Table 2:** **Table2.** Data evaluated from TGA measurements.

Sample	Stage	T_5%_ (°C)	T_max_ (°C)	m (%)	T_endset_ (°C)	W_rez_ (%)
4	I II III	328 – –	318 413 471	6.08 74.49 9.08	326 437 487	10.33
3	I II III	323 – –	322 414 473	7.88 70.84 9.51	335 437 488	11.43
2	I II III	309 – –	336 416 476	13.58 61.32 14.46	345 439 489	10.84
1	I II	445 –	441 494	8.25 76.46	463 503	15.19

T_5%_—temperature corresponding to 5% mass loss; T_max_—temperature corresponding to the maximum rate of decomposition evaluated from the peaks of the DTG curves; T_endset_—endset temperature of thermal degradation corresponding to each stage; m—mass loss per corresponding stage; W_rez_—percentage of residue remained at the end of thermal degradation (700 °C).

**Table 3:** Bacterial contact-killing efficacy determined by Japanese industrial standard JIS Z2801:2000 against: *S. aureus* and *E. coli*.

Sample	R factor	
	*S. aureus*	*E. coli*
1	4.3	4.7
2	5.8	4.9
3	5.6	5.8
4	5.7	5.9
